# Using Autobiography a Pedagogical tool in Pharmacology: Traversing the Un-explored

**DOI:** 10.15694/mep.2020.000063.1

**Published:** 2020-04-03

**Authors:** Anuradha Joshi, Haryax Pathak, Dinesh Badyal

**Affiliations:** 1 Pramukh Swami Medical College; 2 Christian Medical College

**Keywords:** Teaching-Learning, Autobiography, Pharmacology, Pedagogical Tools

## Abstract

This article was migrated. The article was marked as recommended.

In today’s era, medical teaching is becoming increasingly complex and challenging. Out of all subjects in the medical curriculum, Pharmacology is one of the major disciplines undergoing constant development and advancement. A strong foundation of pharmacological knowledge is needed to help students improve upon their understanding and thereby manage clinical conditions in a more effective manner. Unfortunately, Pharmacology is perceived as a dry and volatile subject by medical students. This calls for a need to explore various pedagogical tools at medical schools, so as to best identify methods effective for active learning. Efforts need to be directed towards the objective of making Pharmacology teaching interesting and learner-centered by formulating creative and innovative teaching-learning modules in and outside the classrooms. Learner-centered teaching can foster students’ interest in the subject and contribute to knowledge acquisition as well as future application. In context to this, the current piece explores and addresses the application of “Autobiography of drugs” as one of the pedagogical tools in the subject of Pharmacology.

## Introduction

Pharmacology, like any other branch in medical sciences, is an ever-changing subject, included in the third, fourth and fifth semesters of the medical curriculum in India ( Sharma and Srinivas, 2018). ThoughPharmacology is one of the essential subjects in medicine, most of the medical students perceive it as a dry and volatile subject ( Achike, 2010). New educational insights and scientific developments in the discipline, must prompt pharmacology teachers to design novel ways of teaching-learning methods ( Garg, Rataboli and Muchandi, 2004). In context to this, attempts have been made by teachers to make Pharmacology teaching more interesting and relevant ( Joshi and Kalam, 2013; Joshi *et al.,* 2016). Moreover amidst the fast-paced, technology-driven world, keeping students engaged and excited about their course material can be a real struggle for teachers ( Joshi, 2018). Also, it is a well-known fact that students learn and retain the subject better when they are actively involved in the process of learning (Prasad, Kudthni and Santhosh, 2016; Joshi *et al*., 2016). As teachers in this field, we need to galvanize our efforts towards creating interest in this subject by making it livelier and riveting.

Literature search reports use of Autobiography as an interesting and engaging tool in medical as well as non-medical subjects (Duran *et al*., 2013; Mathibe, 2007). Further exploration reveals that Autobiography can serve as one of the useful teaching-learning pedagogical tools in Pharmacology too! ( Joshi and Ganjiwale, 2015). The need of the hour is to foster interdisciplinary creative pedagogies in the field of education ( Santos, Figueiredo, and Vieira, 2019; Mathur, 2004; Aleinikov, 2013). The current opinion piece explores and simultaneously addresses the use of “Autobiography of drugs” as one of the effective, pedagogical tools to generate interest and active learning in Pharmacology.

## What is Autobiography?

The term ‘Autobiography’ is defined as “(autos self +bios life + graphein to write, *Greek word*)” i.e. a self-written account of the life of oneself. Autobiography is one of the most popular genres introduced in early American literature in the year 1760, exhibited in one of the landmark books written by William Andrews on African American autobiography, “ *To Tell a Free Story”* ( Andrews, 1988). The word “Autobiography” was first used critically by William Taylor in 1797 in an English periodical “The Monthly Review” ( Good, 1981). However, its next recorded use was in its present sense, by Robert Southey in 1809, a prolific writer and commentator, who introduced and popularised several words in the English language ( Good, 1981).

Since all the autobiographical works are by nature subjective, they are a type of experiential narrative which mainly involves narrating one’s story.Everybody has a story to tell, whether your life has been full of accomplishments or littered with hurdles, you have a story you want to share with the world. Autobiographical knowledge usually comprises of providing information on “what the self is”, “what the self was”, and “what the self can be” ( Conway, 2005; Conway and Pleydell-Pearce, 2000). Autobiographies help depict life stories and personal experiences in a riveting manner that allows researchers to deeply understand the way one sees life and construct meaning out of experiences.

Since Autobiography is defined as a story written by a living individual about his or her own life, the authors here opine that the same idea can be adapted to non-living things as well, by using interesting figures of speech like personification, apostrophe, and metaphors. This is strengthened by, interesting literature reports on variable use of metaphors in medical subjects like Pharmacology, Pathology and other para-clinical subjects ( Khilnani, Khilnani and Thaddanee, 2016; Masukume and Zumla, 2012; Khilnani, Khilnani and Thaddanee, 2017). Further, exploration reports use of this concept in clinical practice like a narration of feelings of a disease which have been depicted beautifully in a book titled “Autobiography of a Disease” by Patrick Anderson. Patrick Anderson suddenly falls gravely ill, as his leg is infected with bacteria that in turn infects his bone marrow. Consequently, he undergoes a dozen surgeries in an attempt to remove the bacteria and save his lower limb. Anderson’s book is daring and creative in its attempt to recreate the chaos of illness experience. In sharing the page with bacteria as a fellow narrator, Anderson presents himself not as the victim of disease but as a co-partner with bacteria. The latter lives within Anderson, with his body and therefore is a part of him. Thus, the story of his trauma is told capriciously from three perspectives i.e. from bacteria in his bones, his mother, and himself, in the third-person. This innovative and creative narrative challenges the limitations of the genre of illness memoirs ( Frank, 2017).

Another very good example where this concept has been adopted is in a popular science fiction book titled “Genome” authored by Matt Ridley whereby the author picks up a newly discovered gene from each of 23 pairs of chromosomes and tells the story of chromosome in form of an Autobiography (Ridley, 2000). Matt Ridley himself was inspired to adopt this model from one of the classic novels titled ‘The Periodic Table" authored by Primo Levi’s. ‘The Periodic Table’ depicts a vintage collection of short autobiographical episodes of the author’s experiences as a Jewish-Italian doctoral-level chemist, named after elements of the periodic table ( Levi, 1984).

Another sensitive example of using Autobiography is reported way back in 2009, by Hacking ( Hacking, 2009). The author documents the experiences of autism in the form of narratives which are not just stories or histories describing a given reality, they are indeed creating the language to describe the experience of autism. At the same time, there is a repository of published material on the use of Autobiography as a pedagogical tool in non-medical branches like social, behavioural and political sciences, considering how the ‘I’ interacts with the lives of others (Harger, Brent, and Tim, 2008; Thornton, 2008; Brockmeier, 1997).

## Application of Autobiography as a Pedagogical tool in Pharmacology

Using the concepts of Autobiography in Pharmacology focusses on narrating the life experiences of a non-living substance like a drug in the form of a story in front of the medical world. The whole concept revolves around giving life to an inanimate substance. Thus giving “drug” a voice, a perception, a personality, and a character. Accordingly, itis a kind of learning in which one writes the experiences of a drug as vividly and creatively as one can! In the process, Autobiographies help unveil the characteristic sojourn, the twists and turns in the life of a drug. Associating life and imparting soul to drugs can be made creative and interesting by using various figures of speech or rhetorical figures in abundance ( Khilnani *et al.,* 2016). Autobiography of drugs is one type of a non-conventional pedagogical tool that makes the teaching-learning process captivating interesting, creative ( Joshi and Ganjiwale, 2015), engaging and conceptual. All this blended together fosters active learning ( McCoy *et al.,* 2018). Students also tend to remember important drug details in an interesting manner. While focussing on the contents of Autobiography, one can think about the different periods in the life of drug e.g. historical aspects of drug development, its nature and source, systemic effects including mechanism of action, pharmacokinetics, pharmacodynamics, applied aspects, and adverse drug effects.

A thorough literature search, reveals the use of Autobiography by various educators as a pedagogical tool e.g.in nursing Mathibe ( Mathibe, 2007) has used Lance Armstrong’s autobiography as a teaching tool to teach cytotoxic drugs ( Armstrong, 2001). This, in turn, enhanced students’ interest, participation, and interaction. Statistics suggest that (80%) students found the teaching interesting while 84% felt there was an improvement in their pharmacological knowledge. Autobiographical approaches have also been used as a research methodology tool as a means to illuminate nurses’ experiences and lifelong learning ( Howatson-Jones, 2011). Since Autobiographies are essentially meaningful stories, anything heard in the form of a story is understood and retained better. A very good example of this is depicted in one of the recently published opinion pieces on the confessions of a depressed mind by a surgeon at Michigan Medical School ( Gupta, 2018). Another international study states the role of autobiography as one of the educational techniques in teaching and learning regarding the treatment of diabetes to patients, their families, and relatives. The authors emphasize on application of different educational techniques, one of them being autobiographies, to teach an individual, group or virtual education ( Valverde, Vidal and Jansa, 2012).

Few Pharmacology teachers at an Indian medical school also attempted using Autobiography as one of the teaching-learning tools in Pharmacology ( Joshi and Ganjiwale, 2015), whereby Pharmacology teachers prepared a brief description of drugs in form of autobiographies, using authentic textbooks and reputed journals. Each autobiography consisted of 100- 250 words. In addition, the content was internally as well as externally validated for its appropriateness. Drug description, consisted of hidden clues for the main question i.e. “Who am I”? Also, some triggers were added as an extension to the main question as mentioned below in example no.2 At the same time teacher facilitated the students, discussing the ‘how’ and “why” of answers, to ensure proper learning, thus acting as a “guide by side” rather than “sage by the stage” ( Rege, 2020). “Autobiography of Drugs” was taken as a positive learning experience by students. Authors reported that Autobiography sessions in Pharmacology can be one of the interesting, interactive and creative ways to learn Pharmacology. They also reported that it “triggers imagination”, “helps in better retention of facts”, and that it has “changed their perception regarding Pharmacology learning”. However, they also opined that this is not a stand-alone method for teaching Pharmacology ( Joshi and Ganjiwale, 2015). The reason being learners likely require foundational knowledge to be able to come to conclusions and critically evaluate their pharmacological concepts. Such researches and concepts are still in a nascent stage at various medical colleges, similar studies need to be conducted by Pharmacology teachers in medical colleges to judge its utility as a pedagogical tool.

In another yet interesting study, the Autobiography module was tried in one of the medical colleges in central India ( Tripathi *et al.,* 2015). Herein the “drug’s autobiography” was presented to medical students based on its pharmacokinetics, pharmacodynamics and adverse effects respectively. Following this student had to identify the drugs giving a justification. Students perceived the benefits of active learning strategies in helping them gain clarity on the topic, arousing intellectual curiosity, promoting student interaction and yielding effective learning. A recent review on the status of educational research in India also states that Autobiography of Drugs can foster interest in the subject and help knowledge retention in an interesting way in the subject of Pharmacology (Rege and Tripathi, 2018).

## How to use Autobiography as a Pedagogical tool in Pharmacology

Autobiographies can be used in two ways in Pharmacology classes, first as a part of learner/student-centric activity wherein teachers can ask the students to prepare brief autobiographies in form of experiential narratives or autobiographical essays (unlimited words) of important drugs during free slots in the medical curriculum. So, when in lecture classes, students narrate such pieces, it serves as a good way of breaking the academic monotony and fosters their interest in the subject. While in the second-place teachers can take initiative to prepare brief Autobiographies of drugs (word limit 200- 250 words) and use them as set induction before starting a large classroom lecture or as questions/triggers during revision/ tutorials in Pharmacology. In both cases, one can either start with a narration of some hallmark interesting features related to the history of drug, drug dose, and some peculiar pharmacokinetic or specific pharmacodynamic features, with its application in clinics, adverse drug effects of drug or cost of various formulations of the drug available in the market. Adding quotes related to the history of discovery and actions of drugs also adds flavour to drug description. By writing an autobiography in the words of the subject itself, the student is actually delving deep into the matter and teaching himself in a manner most conducive to his comfort. Ultimately the main goal of preparing interesting Autobiographies lies in designing them in, such a way as if the drug is speaking about itself to the students. Interestingly, the concept is based on giving medicines an attractive personality, which in-turn fosters interest among students and acts as a useful tool for active learning.

## Examples of Autobiographies

### Example 1: Using autobiography as a student-centric activity

Autobiography of “Amoxicillin”

Friends, Romans, and Countrymen lend me your ears..

“ *To err is human” Thanks to* Alexander Fleming the learned scientist, who committed an error somewhere in the year 1928, leading to the discovery of my great, great grandfather, which is none other than the wonder agent, the megastar of the chemotherapy era the antibiotic “Penicillin” ( Roy, 2011). As medical students, we all are aware of this fortunate stroke of serendipity when Fleming left open a Petri dish contaminated with one of the most dangerous gram-positive organisms, called Staphylococcus aureus. Fleming witnessed the growth of a mould displaying a ring of inhibition surrounding this blue coloured cocci “the notorious beast” as I would call it! The mould discovered was none other than Penicillin. *“Success bells for the credentials of my real discovery didn’t ring till, the year 1940. With the joint efforts of the duo, Howard Florey and Ernst Chain, Penicillin was proven safe for humankind and sent for mass production. Thus, in the year 1945 Fleming, Florey and Chain shared the Nobel Prize in Medicine for discovering my dear grandfather. All thanks to the trio!”*

So, friends, just like Parle-G is the ancestor of all biscuits, Penicillin-G is the ancestor of all Penicillins. Let me highlight some core characteristics of my grandfather. My great grandfather consisted of a β-lactam ring, so it was called Beta-lactam antibiotic. β-lactam here depicts the Beta-lactam ring which is actually lethal to cell walls of mainly the gram-positive bacteria ( Deck and Winston, 2012). Unfortunately, in due course of time, the bacteria became resistant. And so, after a lot of research, scientists discovered, or rather deliberately synthesized my father, Ampicillin, in the year 1961. Turns out, all they did was to add an “Amino group”, making him an Aminopenicillin. But it worked wonders. Dad instantly became famous far and wide. Sadly, the enemy grew even stronger and my dad also succumbed! Robust efforts were directed towards making a stronger Beta-lactam antibiotic by adding an extra “hydroxyl” group to my dad and yes it did magic! And so, I was born in 1972, developed by a competent Pharmaceutical by name of Beecham Research Laboratories. Hello, friends, I am “AMOXICILLIN”.

Now that we know my history, let’s see what I do against infectious organisms only.

Oh yeah,

I treat the infectious foci; I have an affinity for the cocci,

Be it gram-positive Strepto, Staphylo, Pneumo or gram-negative Entero,

I kill them all, in vivo..

Oh yeah,

Be it an ulcer peptic, or diarrhoea cryptic,

I’m good against H. pylori, and even Shigella and E. coli..

Oh yeah,

If you get gonorrhoea or meningitis, let’s say the culprit Neisseria is,

I’ll make sure I don’t miss, lest you fall down an abyss..

Oh yeah

If you get an antimicrobial-resistant condition

To make me stronger you can add me with Clavulanic acid too!

As a drug, “I am bioavailable and stable orally. I achieve plasma levels of 4-8 mcg/ ml after an oral dose of 500 mg. I can readily withstand the torture of gastric acid and unlike my dad, food doesn’t interfere with me in my absorption. Approximately 60% of my orally administered dose is excreted by renal route within 6 to 8 hours, therefore please remember to adjust my dose in case of any renal dysfunctions”.

“Everyone needs a partner in their life, this I realized when I started feeling alone and weaker too. My dad, Ampicillin had a partner, Sulbactam. So, do I. My partner is Clavulanic acid. Both Sulbactam and Clavulanic acid are “Beta-lactamase inhibitors” (Sharma and Sharma, 2012). Now you might wonder what are “Beta-lactamase inhibitors, you see, the resistant strains produce an enzyme called Beta-lactamase which renders my Beta-lactam ring useless. For this, if you combine me with Clavulanic acid, this problem just vanishes. So, I and Clavulanic acid were fixed in an FDC (Fixed Dose Combination) by the renowned matchmakers of Pharmaceutical companies. Once again thanks to Beecham Research lab, UK. What synergism! Kudos to this partnership! Inhibition of enzyme β- lactamase, allows us to work smoothly against few resistant bacteria as well. This undoubtedly has given me a new life! Regarding my doses, I’m sold in the market as tablets and capsules in doses of 250/500 mg and as injection too with 125 mg of Clavulanic acid. In adults, I am generally administered as 250-500 mg four times a day. I do go easy on kids 20-40 mg/kg/ day in 3 doses. I am safe in pregnancy but DO NOT use me during breastfeeding”.

Finally, friends, what is life without mischief? I do not worship Loki (you know, the God of Mischief), but I do play little tricks !! Remember every great antibiotic possessing high efficacy and good antimicrobial spectrum, is accompanied by some adverse drug reactions. “I may sometimes cause nausea, vomiting, headache, oral thrush or stomach-ache. Unlike my father, I have fewer propensities to cause diarrhoea, a good relief to share. Maybe some rashes. When things get out of hand, I may have serious side effects like urinary problems, seizures, bloody diarrhoea, and rarely anaphylactic reactions. Oh! how my heart aches when I see quacks prescribing me irrationally. The reason being they are unaware of hazards of irrational prescribing and the toll it takes on patients once I will be declared resistant”.

Well, that’s pretty much everything about me, I am currently one of the most favoured agents commonly used by physicians, paediatricians, surgeons, and gynaecologists singly or in combination with Clavulanic acid. **What about you? Why should you add me with Clavulanic acid?**

### Example 2. Using Autobiography as a trigger to identify the drug

**Autobiography of a drug (Pharmacovigilance**)

Hello everyone! I was one of the first COX-2 inhibitors (Cyclooxygenase enzyme) introduced by a Pharmaceutical company, way back in the year 1999. I was developed especially to favour inhibition of COX-2, but based on an interim analysis of data from Adenomatous Poly Prevention; my adverse drug profile reveals a significant increase in the incidence of thromboembolic events which led to my withdrawal from the market worldwide ( Burke, Smyth and Fitz, 2006).

Trigger questions:

Who am I?

Ans: Rofecoxib

In which year I was withdrawn and by which pharmaceutical company?

Ans: Year 2004, MERK

Can you justify why I can be undesirable in patients at cardiovascular risk?

In the past, selective inhibitors of Cyclooxygenase-2 (COX-2) were basically designed to minimize gastrointestinal complications of traditional Non-Steroidal Anti-inflammatory Drugs (NSAIDs) attributed to the suppression of COX-1-derived prostanoids. Selective COX-2 inhibitors (Coxibs) like Rofecoxib, are effective anti-inflammatory and analgesic drugs. However, recently it has become apparent that some Coxibs increase the risk of serious cardiovascular events, including myocardial infarction and stroke due to concern about an inherent atherothrombotic risk of this class of drugs. COX-2 is regarded as an inducible enzyme involved in the pathophysiology of inflammation and pain. In the cardiovascular system, COX-2 has also been associated with pro-inflammatory/pro-atherogenic stages, due to its up-regulation in monocyte-derived macrophages present in atherosclerotic lesions. COX-2 is “constitutively” expressed in some tissues, among them in the vascular endothelium, where COX-2-derived prostanoids, especially prostacyclin (PGI 2), contribute to the maintenance of vascular homeostasis and integrity. As a result, chronic COX-2 inhibition may have undesirable effects in patients at cardiovascular risk (Martinez and Badimon, 2007).

## Conclusions

Currently, the Indian medical education system is undergoing an exemplar transition in emphasizing on student-centric activities as against conventional teaching. Among the many challenges medical educators face today is the need to make the teaching-learning process captivating, interesting, creative, engaging and meaningful. Creating a classroom environment with differentiated instructional pieces and multifaceted strategies motivates the students and encourages a conceptual understanding of the content ( Chapman, 2009). Bringing changes in the teaching-learning process will, in turn, prepare students to be innovative and will foster non-traditional divergent thinking among students ( King, 1987). What is needed now is transition and reformation in the way teachers teach. This requires an alteration in skill and attitude of teaching by designing creative teaching modules which lead to the creation of significant learning environments. The teachers of today need to develop more effective modes of teaching to incite enthusiasm and creativity among their students ( Joshi and Kalam, 2013). In context to this, autobiography modules can help personalize and deepen the quality of learning and help learners to integrate the material of learning. Since creativity and innovation in education are not just an opportunity but a necessity, pharmacology needs to develop pedagogical tools both in and outside classrooms to make teaching-learning a riveting experience ( Fjortoft, Gettig and Verdone, 2018). Till date concept of Autobiography of Drugs has been used by a few school teachers, pharmacology teachers, as well as a few physiotherapy and pharmacy teachers in India. It has been presented in various international, national and state conferences.

## Take Home Messages

• Writing or narrating autobiography of drugs is like adding a zing to the life of a drug. It is one of those captivating moments in the life of a drug, which helps readers imagine and visualize the sojourn of drugs, in human bodies.
• Autobiography appears to be a promising pedagogical tool that can add a new dimension in the field of pedagogical management, curricular innovations, integration, and interdisciplinary relationships.
• Reading autobiographies of drugs can help students engage better, learn, understand and remember the content in an interesting way that is not visible in traditional teaching.
• Medical teachers should try novel teaching-learning methods and attempt to conduct educational research in relation to one’s setting.
• Educators should consider incorporating learning interventions, even though the strength of such recommendations still needs reinforcement by further research.

## Notes On Contributors

**Dr. Anuradha Joshi**, MBBS, MSc. (Medical Pharmacology), Ph.D. Pharmacology, Professor, Department of Pharmacology, Pramukhswami Medical College, Fellowship in Medical Education (CMCL FAIMER 2016: Foundation for Advancement of International Medical Education), Faculty Advisor: CMCL FAIMER 2018, Advance Course in Medical Education (ACME 2014), Resource faculty at Nodal Centre for Faculty Development, Medical Education Unit, Member AHPE (Academy of Health Profession Educators, India), Member IPS (Indian Society Pharmacology), ISOP (Indian Society Pharmacovigilance), Gokul Nagar, Karamsad (388325), Gujarat (India). ORCiD: https://orcid.org/0000-0001-5389-785X

**Dr. Haryax Pathak**, Intern, Pramukh Swami Medical College, Karamsad, Gujarat, India. ORCiD: https://orcid.org/0000-0003-3683-3672

**Dr. Dinesh Badyal,** MD, Dip. In Clinical Research, MHPE (UK), Fellow FAIMER (Philadelphia), Professor and Head, Department of Pharmacology, Professor, Department of Medical Education, Christian Medical College, Ludhiana-141008, India, Program Co-Director, CMCL-FAIMER Regional Institute, Co-convener, MCI Nodal Center for Faculty Development, Senior Editor, Indian Journal of Pharmacology, Member, Core Training Panel, Pharmacovigilance Program of India, Coordinator, Pharmacovigilance Center, CMC.

## Declarations

The author has declared that there are no conflicts of interest.

## Ethics Statement

There has been no active intervention on any living being nor was any private information collected. This is purely an opinion piece.

## External Funding

This article has not had any External Funding
