# Supplementary material for: Using Autobiography a Pedagogical tool in Pharmacology: Traversing the Un-explored
Source: MedEdPublish (2016). 2020 Apr 3;9:63. [Version 1] doi: 10.15694/mep.2020.000063.1 (PMC10697585; doi:10.15694/mep.2020.000063.1)
Supplement: Peer review history

# Reviewer report: Reviewer response for version 1

**DOI:** 10.21956/mep.19987.r30413

**Published:** 2020-04-05

**Authors:** P Ravi Shankar

**Affiliations:** 1 American International Medical University

This review has been migrated. The reviewer awarded 5 stars out of 5 I enjoyed reading this well-written and beautifully structured article. I am glad to know that innovative teaching-learning methods are being employed to learn pharmacology. Autobiography can be an effective method and enables students to think and learn from a different perspective. The authors have provided many examples of the use of autobiography in medical literature. It will be nice if they can provide some examples of the use of autobiography as a method of teaching medical students. I am also happy to note that they have also included the history of medicine where relevant going by the example of Sir Alexander Fleming in the case study on amoxicillin. The example of amoxicillin has been very well-written and is interesting. My only concern is about the depiction of the combination of amoxicillin with clavulanic acid. I do agree it is a therapeutic advance but its use is limited to infections caused by beta-lactamase producing bacteria. The combination has been widely used and may be overused in conditions where it is not indicated. It will be nice if this issue can be addressed in the autobiography. The authors can consider allowing some of their autobiographies to be used by other teachers to help students learn pharmacology and deposit some of them on a publicly accessible database. This interesting article will be of broad interest.

Reviewer Expertise:

NA

No decision status is available

# Reviewer report: Reviewer response for version 1

**DOI:** 10.21956/mep.19987.r30414

**Published:** 2020-04-03

**Authors:** Manish Khazane

**Affiliations:** 1 SRM UNIVERSITY,RAMAPURAM,CHENNAI.

This review has been migrated. The reviewer awarded 5 stars out of 5 Truly, a refreshing read. My sincere thanks to the authors for putting up this wonderful article. Amongst all the chaos, the use of autobiography as a learning tool brings about an interesting twist to the teaching methods. As they say, change is the only constant. It would be very interesting to see, how such new methods can be collaborated with technology and mass replicated in the Indian medical education system.

Reviewer Expertise:

NA

No decision status is available
